# Burden of multiple myeloma in Asian countries from 1990 to 2021 and trend prediction for 2050: an analysis based on the Global Burden of Disease Study 2021

**DOI:** 10.3389/fmed.2025.1696768

**Published:** 2025-12-18

**Authors:** Chengjun Hu, Weifeng Chen, Ping Zhang, Tongping Shen, Yu Zhao

**Affiliations:** 1 Department of Hematology, The Affiliated Jiangyin Hospital of Nantong University, Jiangyin, Jiangsu, China; 2 School of Information Engineering, Anhui University of Chinese Medicine, Hefei, Anhui, China

**Keywords:** Asia, burden of disease, Human Development Index, multiple myeloma, predictions

## Abstract

**Background:**

Multiple myeloma (MM) poses a growing challenge to global public health, with regional disparities in disease burden becoming increasingly prominent. As the most populous continent, Asia is undergoing rapid demographic and socioeconomic transitions, and plays a critical role in shaping the evolutionary trajectory of the global MM burden. This study aims to assess the disease burden of multiple myeloma in Asian countries from 1990 to 2021.

**Methods:**

The data of this study were derived from the Global Burden of Disease Study 2021 database. We conducted a systematic analysis of the disease burden of MM in Asia from 1990 to 2021, covering four core dimensions: incidence, prevalence, mortality, and disability-adjusted life years (DALYs). To fully present the complexity of the disease burden, we also performed multiple analytical approaches, including trend analysis, gender-stratified analysis, regional comparative analysis, and ARIMA model prediction analysis.

**Results:**

In 2021, incident cases of MM in Asia accounted for 35.8% of the global total, prevalent cases for 34.2%, deaths for 36.3%, and DALYs for 39.7%, making Asia a key contributing region to the global MM disease burden. From 1990 to 2021, the growth rates of the above indicators in Asia were significantly higher than the global average. Moreover, ARIMA model projections indicated that Asia’s MM burden would follow a “first increase, then stabilize” trajectory by 2050, with a more prominent growth magnitude and higher prediction uncertainty observed in men. This disparity may reflect a combination of metabolic, lifestyle, and genetic factors East Asia and South Asia emerged as the core regions bearing the MM burden, while Central Asia exhibited the fastest growth rate in burden. High-income regions had higher MM diagnosis rates (reducing underreporting), whereas low-income regions faced significant underestimation of the actual burden due to limited diagnostic capacity. Additionally, as socioeconomic status decreased, the average age at death of MM patients advanced.

**Conclusion:**

The study confirms that Asia has become the primary driving region for the growth of the global MM burden, and its burden pattern is influenced by significant gender disparities, regional disparities, and socioeconomic disparities.

## 1 Introduction

Multiple myeloma (MM) is the second most prevalent hematologic disease among plasma cell malignancies and is typically characterized by abnormally elevated serum monoclonal immunoglobulins. If left untreated, MM often causes severe end-organ damage, with clinical manifestations of osteolytic bone lesions, anemia, hypercalcemia, renal failure, and abnormal accumulation of malignant plasma cells (1). According to statistics, there are approximately 140,000 new cases of MM patients worldwide each year, with an age-standardized incidence rate of 2.1 cases per 100,000 people. With the aging of the population, its incidence rate has shown a continuous upward trend (2).

Although the exact etiology of MM has not been fully elucidated, genetic factors are believed to play a significant role in its pathogenesis. Studies have shown that immediate family members of MM patients have a significantly higher risk of developing the disease than the general population (3). Additionally, multiple risk factors are strongly linked to the development of MM. Environmental exposures (radiation and specific chemicals) (4) and lifestyle factors, such as high body mass index (BMI) (5), have also been shown to promote the development of MM.

Currently, monoclonal gammopathy of undetermined significance (MGUS) and smoldering multiple myeloma (SMM) have been identified as precursor states of MM. Therefore, early recognition and intervention of MGUS and SMM are important for slowing the progression of MM, prolonging patient survival, and reducing end-organ damage. It has been shown that patients with MGUS have a lower incidence of end-organ disease when progressing to MM, which may be attributed to the intervention of early treatment and support services (6). However, the asymptomatic nature of MGUS and SMM poses a challenge for early clinical intervention (7). Current consensus guidelines recommend ongoing surveillance of patients with MGUS to assess their risk of progression to MM, but how to optimize screening strategies for MGUS, SMM, and MM in high-risk populations (African Americans, elderly patients, and obese patients) remains key to improving prognosis (8).

In recent years, the incidence and prevalence of MM have shown a significant upward trend globally (9, 10). Epidemiologic data show that there are substantial geographic variations in the incidence of MM, with higher incidence rates generally found in high-income countries (11) and relatively lower incidence rates found in Asia and sub-Saharan Africa, a phenomenon that highlights the influence of genetic factors, environmental exposures, and healthcare resource distribution on the epidemiologic characteristics of the disease.

In the therapeutic area, a variety of non-targeted therapeutic agents have been approved for the clinical management of multiple myeloma over the past few decades. These include alkylating agents (12), corticosteroids (13), immunomodulatory imide drugs (IMiD) (14), proteasome inhibitors (PI) (15), histone deacetylase (iHDAC) inhibitors (16), and XPO1 inhibitors (17). Although these therapeutic regimens have significantly prolonged patient survival by inhibiting myeloma cell proliferation and controlling disease progression, they are often accompanied by severe adverse effects due to their nonspecific mechanisms of action. The rapid proliferative nature of myeloma cells and their difficulty in complete eradication is one of the primary reasons multiple myeloma is difficult to eradicate. While killing tumor cells, non-targeted drugs also cause damage to normal tissues, triggering a series of adverse reactions, including gastrointestinal reactions (nausea, vomiting, diarrhea, and constipation) (18), bone marrow suppression (manifested as leukopenia and thrombocytopenia), alopecia, anemia, hepatic and renal function impairment, and increased risk of infection (19, 20).

Notably, in low- and middle-income countries with limited healthcare resources, patients’ median survival tends to be significantly shorter, reflecting significant differences in treatment outcomes globally.

Although the expected survival of elderly patients over 65 is relatively short, autologous stem cell transplantation, as a routine treatment in developed countries (e.g., Europe, Australia, and North America) (21), has shown favorable treatment outcomes even in elderly patients over 75 (22).

Additionally, social determinants have a significant impact on the prognosis and survival outcomes of MM patients. Social factors, including socioeconomic status, level of healthcare coverage, education, employment status, and community support, can significantly improve patients’ clinical outcomes (23). Ensuring access to healthcare, including removing financial barriers through systematic measures, is essential to guarantee access to novel treatment options and supportive care for vulnerable groups (24). For example, dietary interventions, particularly nutritional programs based on plant-based diets, have been shown to improve metabolic and microbiome biomarkers in patients with MGUS and SMM, underscoring the importance of enhancing nutritional access for MM patients in food deserts (25).

Multiple myeloma not only imposes a heavy economic burden on global health systems, but the cost of its treatment also varies significantly between regions, reflecting the uneven accessibility and distribution of healthcare resources. In addition, MM can cause a variety of extramedullary lesions that involve multiple organ systems, including the immune, nervous, and musculoskeletal systems. These secondary damages significantly increase the direct and indirect treatment costs, further exacerbating the socioeconomic burden, especially in resource-poor areas.

While precision medicine approaches and novel targeted therapies have significantly improved survival rates in developed healthcare systems, a therapeutic disparity persists across resource-stratified regions, particularly in low- and middle-income countries, as documented in recent health disparity reports (26). This geographical inequity in care accessibility underscores the imperative for establishing multinational collaborative frameworks to standardize management protocols for MM. Despite numerous investigations characterizing MM’s pathophysiological determinants and socioeconomic impacts, persistent gaps remain in contemporary multinational epidemiological surveillance, particularly regarding temporal trends in treatment accessibility and outcome disparities.

The GBD 2021 study provides a comprehensive overview of MM’s current global epidemiologic status. Based on this, this study aims to utilize the GBD 2021 database to systematically characterize the global epidemiological profile and disease burden of MM, predict future trends, analyze risk factors, and reveal differences between regions and countries through geographic stratification and temporal trend analyses, thereby proposing targeted prevention strategies. In addition, this study will examine the key drivers of MM disease burden, including population aging, improved diagnostics, and advancements in treatment. Through this systematic study, we aim to provide a scientific basis for MM prevention, diagnosis, and treatment strategies globally, as well as a reference for low- and middle-income countries to formulate policies that reduce the MM disease burden.

## 2 Materials and methods

### 2.1 Data sources

Epidemiologic data on multiple myeloma were obtained through the Global Health Data Exchange (GHDx) query tool.^1^ The tool offers a standardized data extraction function that meets the researcher’s needs for specific disease burden indicators.

To ensure the comprehensiveness and representativeness of the data, we extracted multiple myeloma-related statistics, including incidence, mortality, and DALYs, from the GBD database from 1990 to 2021. These indicators provide a comprehensive picture of how the disease affects the population’s health.

In the analysis, the Socio-demographic Index (SDI) is used as an essential indicator of a region’s level of social development, which is a composite score calculated by standardizing the total fertility rate, the average educational attainment of the population over 15 years of age and the ranking of per capita income, with a value ranging between 0 and 1. Based on the level of the SDI, countries and regions of the world are categorized into five classes: low, medium-low, medium, medium-high, and high SDI regions. This categorization helps to identify differences in disease burden between regions at different stages of social development.

Geographic characteristics are one of the key factors influencing disease distribution. Therefore, we analyzed 48 Asian countries, covering representative regions such as East Asia and South Asia. This division method can more elaborately reflect the heterogeneity of the epidemiological characteristics of multiple myeloma across Asia. East Asia includes China, Japan, the Republic of Korea, the Democratic People’s Republic of Korea, and Mongolia. Southeast Asia comprises Indonesia, Thailand, the Philippines, Vietnam, Myanmar, Cambodia, the Lao People’s Democratic Republic, Malaysia, Singapore, Brunei Darussalam, and Timor-Leste. South Asia encompasses India, Pakistan, Bangladesh, Sri Lanka, Nepal, Bhutan, the Maldives, and Afghanistan. West Asia comprises Türkiye, the Islamic Republic of Iran, Iraq, Saudi Arabia, Yemen, Oman, the United Arab Emirates, Qatar, Kuwait, Bahrain, Lebanon, Jordan, the Syrian Arab Republic, Palestine, Israel, Cyprus, Armenia, Azerbaijan, and Georgia. Central Asia includes Kazakhstan, Uzbekistan, Turkmenistan, Tajikistan, and Kyrgyzstan.

The methodology used in this study is consistent with the overall GBD 2021 methodology (27). In addition, the Human Development Index (HDI), a key measure of the level and availability of health resources, was included in the analytic framework, as published by the United Nations Development Programme.^2^

The Age-Standardized Rate (ASR) was the core analytical indicator in the data processing process. The ASR is weighted to eliminate the interference of differences in age structure and population size across regions when estimating the burden of disease (28), thereby improving the validity and comparability of comparative analyses.

### 2.2 Statistical analysis

We quantified trends in the burden of multiple myeloma using EAPC to describe the magnitude of changes in ASR trends, including ASIR, ASMR, and ASR.

The age-standardized rate is calculated per 100,000 population using the following formula Equation 1:


$$A\,S\,R=\frac{\sum_{i=1}^{A}a_{i}\,w_{i}}{\sum_{i=1}^{A}w_{i}}\times 100 000$$ (1)

Where ASR is the age-standardized rate, *a_i_* is the age-specific rate for the *i*_*th*_ age group, *w_i_* is the number of people in the standard population corresponding to the ith age group, and A is the number of age groups.


y=α+β⁢x+ε (2)

E⁢A⁢P⁢C=100×(e⁢x⁢p⁢(β)-1) (3)

The trajectory of age-standardized rates is assessed by analyzing the Estimated Annual Percentage Change (EAPC), which is calculated through (Equations 2, 3), alongside its 95% confidence interval. A positive EAPC value, coupled with a positive lower limit of the 95% confidence interval, signifies an increasing trend in age-standardized rates. Conversely, a negative EAPC value and a negative 95% confidence interval upper limit indicate a decreasing trend in these rates.

To explore factors influencing EAPC, we used Pearson correlation analysis to estimate the relationship between EAPC and HDI in 2021. We explored the age distribution of multiple myeloma at 5-year intervals and assessed age-specific potential risk factors for various myeloma-related deaths and DALYs.

### 2.3 Autoregressive moving average model

The Autoregressive Integrated Moving Average (ARIMA) model, as an essential time-series forecasting method in GBD research, is primarily used to address the residual variance after the mixed-effects model or spline function has extracted the primary trend. The modeling strategy is based on the fundamental assumptions that the residual series must satisfy a linear relationship, exhibit smooth characteristics, and show no autocorrelation. This is validated by unit root tests (ADF test) and an assessment of the residual autocorrelation plot. In the model comparison, the research team found that, despite the simultaneous testing of nonlinear prediction techniques such as ETS, ARIMA was ultimately selected as the core prediction tool due to its superior amount of bare-pool information (29) and its ability to accurately capture multiple myeloma-specific evolutionary patterns. According to the methodological consensus in international disease prediction, this study employed the model to construct a prediction system for MM incidence trends in various regions of the world by 2050 (30).

### 2.4 Data visualization

We utilized packages including ggmap, ggplot2, tidy, reshape, and RColorBrewer for data visualization, and the dplyr package for data cleaning. The ggmap visualized the MM EAPC from 48 Asian countries. All statistical analyses and visualizations were conducted using R software (version 4.3.0). The burden of disease data for the study in this paper were obtained from GBD 2021, and no ethical material was required (31).

## 3 Results

### 3.1 Global and regional burden of MM

From 1990 to 2021, the global incident cases of MM increased from 55,710 (95% UI: 52,022–59,688) to 148,755 (95% UI: 131,780–162,049), and the ASIR increased from 1.47 per 100,000 persons (95% UI: 1.37–1.57) in 1990 to 1.74 per 100,000 persons (95% UI: 1.54–1.89) in 2021, with an ASIR EAPC of 0.48 (95% CI: 0.36–0.60); Asian incident cases grew by 348.2%, increasing to 53,218 cases (accounting for 35.8% of the global total) in 2021, and Asia’s ASIR EAPC was 1.59 (95% CI: 1.44–1.75, the highest among the four continents), while the Americas saw a declining ASIR with an EAPC of −0.14% (95% CI: −0.25 to -0.03).

**FIGURE 1:**
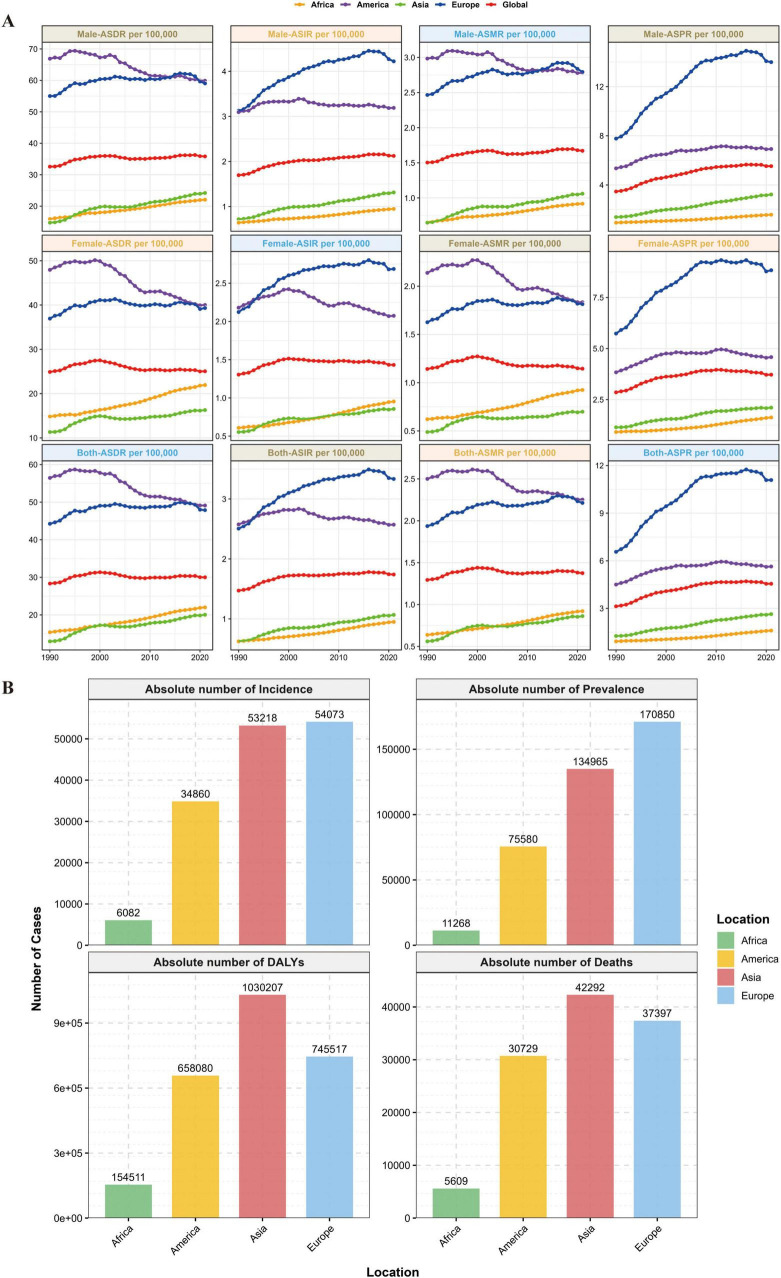
Global burden of disease distribution. **(A)** Global and MM disease burden trends across the four continents. **(B)** 2021 distribution of incidence, prevalence, DALYs, and deaths across the four continents.

**FIGURE 2:**
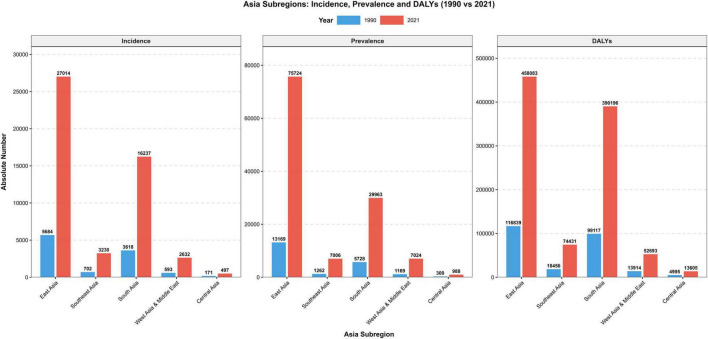
The burden of MM in Asia between 1990 and 2021.

From 1990 to 2021, the global prevalent cases of MM increased from 123,974 (95% UI: 117,342–130,805) to 394,482 (95% UI: 355,593–425,503), with an ASPR EAPC of 1.24 (95% CI: 1.02–1.46); Asian prevalent cases grew by 439.2%, accounting for 34.2% of the global total in 2021, and Asia’s ASPR EAPC was 2.49 (95% CI: 2.34–2.64, the highest among the four continents). The number of global MM deaths in 2021 reached 116,360 (95% UI: 103,079–128,471), with an ASMR EAPC of 0.09 (95% CI: −0.01–0.19), and from 1990 to 2021, global deaths increased by 144.6%; in contrast, Asian deaths grew by 311.3%, accounting for 36.3% of the global total in 2021 (surpassing Europe for the first time), with an ASMR EAPC of 1.18 (95% CI: 0.99–1.36), while the Americas had a declining ASMR with an EAPC of −0.50 (95% CI: −0.59 to −0.41). From 1990 to 2021, the global MM-related DALYs increased from 1,122,517 (95% UI: 1,041,399–1,227,729) to 2,595,595 (95% UI: 2,270,484–2,889,968), with an ASDR EAPC of 0.06 (95% CI: −0.04–0.15); Asian DALYs grew by 280.8%, accounting for 39.7% of the global total in 2021, and Asia’s ASDR EAPC was 1.20 (95% CI: 1.02–1.37, the highest among the four continents), while the Americas saw a declining ASDR with an EAPC of −0.64 (95% CI: −0.73 to −0.55) (see Table 1 and Figures 1, 2).

**TABLE 1:** ASIR, ASPR, ASMR, and ASDR in Global, 1990–2021.

World region	1990		2021		1990–2021	1990		2021		1990–2021
	Incident cases	ASIR per 100,000	Incident cases	ASIR per 100,000	EAPC	Prevalence cases	ASPR per 100,000	Prevalence cases	ASPR per 100,000	EAPC
	No. *10^2^ (95% UI)	No. (95% UI)	No. *10^2^ (95% UI)	No. (95% UI)	No. (95% CI)	No. *10^2^ (95% UI)	No. (95% UI)	No. *10^2^ (95% UI)	No. (95% UI)	No. (95% CI)
Overall	557.1 [520.22–596.88]	1.47 [1.37–1.57]	1487.55 [1317.8–1620.49]	1.74 [1.54–1.89]	0.48 [0.36– 0.6]	1239.74 [1173.42–1308.05]	3.13 [2.96–3.3]	3944.82 [3555.93–4255.03]	4.55 [4.1–4.91]	1.24 [1.02– 1.46]
**Sex**										
Male	284.55 [262.72–311.21]	1.7 [1.57–1.85]	824.54 [714.57–907.36]	2.12 [1.83–2.34]	0.7 [0.59– 0.81]	629.94 [591.32–675.13]	3.47 [3.25–3.71]	2231 [1949.41–2430.94]	5.53 [4.85–6.01]	1.57 [1.35– 1.79]
Female	272.55 [251.98–298.64]	1.3 [1.2–1.43]	663.01 [560.21–752.87]	1.43 [1.21–1.62]	0.2 [0.07– 0.34]	609.81 [569.72–652.6]	2.86 [2.66–3.05]	1713.81 [1466.03–1910.52]	3.72 [3.18–4.15]	0.85 [0.62– 1.08]
**Region**										
Asia	118.74 [101.7–150.2]	0.63 [0.54–0.79]	532.18 [426.14–639.93]	1.07 [0.85–1.28]	1.59 [1.44– 1.75]	250.32 [219.57–302.61]	1.26 [1.11–1.51]	1349.65 [1074.63–1583.18]	2.64 [2.12–3.09]	2.49 [2.34– 2.64]
Africa	17.16 [11.01–21.86]	0.62 [0.4–0.79]	60.82 [39.37–77.07]	0.95 [0.61–1.18]	1.42 [1.38– 1.46]	27.32 [17.62–34.56]	0.92 [0.59–1.17]	112.68 [74.46–140.84]	1.6 [1.05–2]	1.88 [1.79– 1.97]
America	156.79 [149.18–161.39]	2.58 [2.45–2.65]	348.6 [321.16–364.67]	2.57 [2.37–2.69]	–0.14 [–0.25 to −0.03]	274.43 [263.83–284.43]	4.5 [4.33–4.67]	755.8 [708.41–794.51]	5.64 [5.29–5.93]	0.64 [0.46– 0.83]
Europe	262.16 [250.06–271.09]	2.51 [2.39–2.59]	540.73 [489.42–573.75]	3.33 [3.06–3.53]	0.95 [0.78– 1.11]	681.76 [651.73–710.94]	6.56 [6.28–6.84]	1708.5 [1576.45–1810.76]	11.09 [10.3–11.73]	1.77 [1.46– 2.09]
	**1990**		**2021**		**1990–2021**	**1990**		**2021**		**1990–2021**
	**DALYs cases**	**ASDR per 100,000**	**DALYs cases**	**ASDR per 100,000**	**EAPC**	**Deaths cases**	**ASMR per 100,000**	**Deaths cases**	**ASMR per 100,000**	**EAPC**
	No. *10^2^ (95% UI)	**No. (95% UI)**	No. *10^2^ (95% UI)	**No. (95% UI)**	**No. (95% CI)**	No. *10^2^ (95% UI)	**No. (95% UI)**	No. *10^2^ (95% UI)	**No. (95% UI)**	**No. (95% CI)**
Overall	11225.17 [10413.99–12277.29]	28.34 [26.33–30.83]	25955.95 [22704.84–28899.68]	30 [26.22–33.37]	0.06 [–0.04 to 0.15]	475.69 [441.38–514.17]	1.29 [1.2–1.39]	1163.6 [1030.79–1284.71]	1.37 [1.22–1.52]	0.09 [–0.01 to 0.19]
**Sex**										
Male	5923.27 [5324.7–6650.76]	32.57 [29.55–36.21]	14443.23 [12190.73–16147.93]	35.82 [30.46–39.98]	0.22 [0.14– 0.3]	240.77 [219.21–267.21]	1.5 [1.38–1.66]	631.22 [544.4–701.8]	1.67 [1.44–1.86]	0.27 [0.19– 0.35]
Female	5301.9 [4882.38–6013.86]	24.89 [22.94–28.15]	11512.72 [9402.88–13370.62]	25.04 [20.39–29.1]	–0.16 [–0.27 to –0.05]	234.92 [215.74–259.67]	1.14 [1.04–1.26]	532.38 [448.3–608.8]	1.14 [0.96–1.31]	–0.14 [–0.25 to –0.02]
**Region**										
Asia	2705.38 [2247.67–3563.93]	12.96 [10.81–16.76]	10302.07 [8203.76–12757.51]	20.04 [16.03–24.65]	1.2 [1.02– 1.37]	102.83 [86.34–132.71]	0.56 [0.47–0.71]	422.92 [341.12–514.97]	0.86 [0.69–1.05]	1.18 [0.99– 1.36]
Africa	459.65 [295.83–587.12]	15.37 [9.86–19.61]	1545.11 [993.3–1984.47]	22 [14.16–27.81]	1.22 [1.19– 1.25]	16.83 [10.82–21.51]	0.64 [0.41–0.81]	56.09 [36.04–70.39]	0.92 [0.59–1.14]	1.25 [1.22– 1.28]
America	3441.18 [3316.86–3526.11]	56.45 [54.37–57.86]	6580.8 [6180.68–6860.89]	49.11 [46.18–51.17]	–0.64 [–0.73 to –0.55]	151.77 [143.56–156.36]	2.5 [2.36–2.58]	307.29 [280.64–321.62]	2.25 [2.06–2.36]	–0.5 [–0.59 to –0.41]
Europe	4578.01 [4405.93–4705.35]	44.24 [42.62–45.5]	7455.17 [6892.49–7874.33]	47.87[44.56–50.52]	0.22 [0.13– 0.31]	202.55 [192.78–208.84]	1.94 [1.84–2]	373.97 [338.45–397.15]	2.21 [2.02–2.34]	0.41 [0.32– 0.5]

Notably, Asia had the highest EAPC values among the four continents for all four core indicators, ASIR, ASPR, ASMR, and ASDR and these rates were significantly higher than the global average. This confirms that Asia has become the primary driving region for the global growth of the MM burden.

### 3.2 Asian and regional burden of MM

From 1990 to 2021, the MM burden including incidence, prevalence, mortality, and DALYs, increased significantly across all 48 Asian countries, with notable variations among countries and between genders. The MM burden and its growth rate were generally higher in males than in females.

In 2021, China and India ranked among the top in Asia in terms of new MM cases, prevalent cases, deaths, and DALYs, each collectively accounting for over 50% of the total in Asia. In contrast, small island nations such as Timor-Leste and the Maldives had the fewest cases.

Regarding trends, Georgia, Turkmenistan, and Armenia recorded the most prominent increases in the ASIR, ASPR, and ASMR. Only a small number of countries, including Tajikistan, exhibited a declining or slow-growing trend in relevant indicators—for instance, Tajikistan’s ASIR EAPC was –0.66 (95% CI: –0.93 to –0.38), ASPR EAPC was –0.57 (95% CI: –0.88 to –0.26), and ASMR EAPC was –0.70 (95% CI: –0.97 to –0.44). Kuwait also showed a declining ASMR trend with an EAPC of –0.44 (95% CI: –1.32 to 0.35), while Singapore’s ASDR EAPC was –1.74 (95% CI: –1.86 to –1.62) and Tajikistan’s ASDR EAPC was –0.75 (95% CI: –1.04 to –0.46).

By region, East Asia was the subregion with the heaviest MM burden in Asia, with China and South Korea among the top in terms of burden. South Asia’s MM burden grew rapidly, with India ranking second in Asia across multiple indicators. High-income countries in West Asia had relatively high incidence rates but moderate growth, while countries like Georgia and Turkmenistan experienced explosive growth, their EAPC values for key indicators exceeded 6%. Southeast Asia’s burden rose steadily, with Indonesia bearing the heaviest burden in this subregion. Central Asia had a relatively low absolute burden but a fast growth rate; for example, Kyrgyzstan’s ASIR EAPC was 3.37 (95% CI: 2.12–4.63) (see Table 2).

**TABLE 2:** ASDR in 48 Asian countries, 1990–2021.

Country	1990		2021		1990–2021
	DALYs cases	ASDR per 100,000	DALYs cases	ASDR per 100,000	EAPC
	No. *10^2^ (95% UI)	No. (95% UI)	No. *10^2^ (95% UI)	No. (95% UI)	No. (95% CI)
Cyprus	4.28 [3.03–6.36]	55.07 [38.27–81.55]	10.1 [6.48–13.56]	48.47 [31.31–64.99]	–0.17 [–0.37 to 0.02]
Israel	31.06 [28.55–33.57]	63.98 [58.81–69.04]	71.51 [63.87–78.21]	58.31 [52.47–63.56]	–0.34 [–0.55 to –0.12]
Yemen	5.34 [2.08–10.38]	9.94 [3.94–19.61]	18.77 [9.86–32.88]	11.96 [6.41–21.14]	0.85 [0.7–0.99]
Myanmar	14.57 [8.24–29.54]	5.82 [3.31–11.74]	48.62 [29.66–92.8]	9.33 [5.7–18]	1.69 [1.62– 1.76]
Tajikistan	2.34 [1.47–3.63]	7.43 [4.6–11.2]	4.56 [3.01–6.66]	6.11 [4.06–8.88]	–0.75 [–1.04 to –0.46]
Lebanon	12.78 [8.04–18.55]	57.14 [35.92–81.94]	33.87 [23.63–46.47]	57.04 [40.04–78.48]	0.41 [0.23– 0.6]
China	468.54 [320.56–925.93]	5.02 [3.42–9.99]	3383.59 [2136.69–4476.35]	16.12 [10.09–21.35]	3.15 [2.44– 3.87]
Sri Lanka	20.04 [15.57–28]	17.53 [13.59–24.47]	66.17 [39.67–98.16]	23.71 [14.31–35.2]	1.24 [1.06– 1.42]
Philippines	22.85 [15.24–29.59]	6.94 [4.66–9.23]	99.68 [73.74–129.84]	11.07 [8.24–14.66]	1.59 [1.56– 1.63]
Democratic People’s Republic of Korea	13.75 [8.64–24]	7.71 [4.87–13.55]	27.05 [15.35–46.03]	8.04 [4.58–13.74]	0.31 [0.23– 0.39]
Thailand	36.5 [24.1–65.51]	9.57 [6.32–17.21]	153.01 [94.48–288.57]	14.06 [8.76–26.55]	0.67 [0.42– 0.91]
Bhutan	0.41 [0.15–0.68]	15.62 [5.86–26.26]	1.52 [0.76–2.78]	24.58 [12.43–44.49]	1.48 [1.4–1.55]
Republic of Korea	67.5 [43.25–96.64]	21.79 [14.31–32.27]	234.46 [137.94–309.65]	24.74 [14.64–32.59]	0.55 [0.34– 0.76]
Cambodia	3.2 [1.66–5.99]	6.55 [3.41–12.58]	14.91 [8.43–30.12]	11.09 [6.24–22.84]	1.8 [1.61– 2]
Indonesia	71.69 [53.48–126.85]	6.62 [4.9–11.81]	274.51 [179.79–503.95]	10.37 [6.7–19.35]	1.38 [1.33– 1.43]
Afghanistan	9.84 [2.5–21.76]	13.3 [3.41–29.1]	19.07 [7.6–43.22]	16.66 [6.86–35.61]	1.04 [0.74– 1.33]
Turkmenistan	1.81 [1.5–2.19]	7.74 [6.44–9.33]	15.3 [11.39–21.06]	31.83 [23.83–43.57]	6.04 [5.42– 6.66]
India	738.21 [428.55–939.88]	14.78 [8.71–18.84]	2994.44 [2361.3–3978.64]	24.22 [19.08–32.16]	1.46 [1.27– 1.65]
Viet Nam	12.73 [8.8–16.76]	3.11 [2.15–4.09]	67.37 [45.89–93.2]	6.52 [4.42–8.85]	2.59 [2.54– 2.64]
Nepal	11.53 [4.74–18.45]	11.64 [4.88–18.64]	45.2 [22.85–76.35]	18.77 [9.54–31.49]	1.66 [1.38– 1.95]
Uzbekistan	4.83 [3.88–5.96]	3.73 [2.97–4.59]	18.12 [13.58–23.27]	5.81 [4.4–7.42]	1.27 [0.9–1.65]
Bangladesh	71.93 [38.77–107.43]	14.67 [7.95–22.02]	258.72 [151.47–443.44]	18.21 [10.76–31.13]	0.61 [0.5–0.71]
Japan	617.99 [585.88–637.92]	35.89 [33.98–37.07]	933.34 [809.42–1011.31]	25.68 [23.2–27.3]	–1.24 [–1.39 to –1.08]
Malaysia	14.68 [10.7–21.11]	15.08 [11.04–21.89]	65.64 [52.03–94.56]	21.91 [17.37–31.69]	1.17 [1.04– 1.3]
Lao People’s Democratic Republic	1.48 [0.63–3.02]	6.6 [2.85–13.37]	5.14 [2.74–9.98]	10.11 [5.42–19.95]	1.46 [1.39– 1.53]
Saudi Arabia	10.15 [6.38–14.54]	16.04 [10.09–23.01]	63.17 [41.4–86.94]	25.68 [16.38–34.33]	1.63 [1.53– 1.73]
Georgia	5.68 [4.66–6.75]	8.94 [7.35–10.59]	20.72 [17.79–24.01]	38.46 [33.11–44.33]	5.98 [5.2–6.77]
Syrian Arab Republic	5.03 [3.31–7.63]	8.77 [5.8–13.4]	15.9 [9.59–23.66]	11.08 [6.78–16.38]	0.91 [0.76– 1.07]
Kyrgyzstan	1.68 [1.34–2.05]	5.5 [4.42–6.7]	4.87 [3.76–6.14]	8.48 [6.55–10.6]	3.28 [2.21– 4.37]
Brunei Darussalam	0.59 [0.41–0.85]	57.44 [40.06–82.98]	1.75 [1.26–2.32]	48.01 [34.64–62.54]	–0.05 [–0.22 to 0.11]
Maldives	0.11 [0.06–0.17]	11.52 [6.14–16.82]	0.47 [0.33–0.66]	12.64 [8.98–16.96]	0.04 [–0.09 to 0.17]
Pakistan	148.93 [107.91–205.15]	25.54 [18.49–35.21]	535.43 [374.92–773.99]	40.86 [28.8–58.75]	1.32 [1.15– 1.49]
Türkiye	148.77 [96.28–205.97]	40.06 [26.09–55.09]	444.86 [324.18–602.48]	45.96 [33.7–62.25]	0.3 [0.11– 0.49]
Palestine	2.84 [1.68–4.14]	31.24 [18.68–44.99]	10.35 [6.02–13.67]	37.18 [21.86–49.08]	0.59 [0.53– 0.64]
Bahrain	0.75 [0.55–1.07]	38.74 [28.86–55.51]	4.23 [2.4–6.28]	41.25 [24.46–57.9]	–0.02 [–0.18 to 0.14]
Oman	1.6 [0.88–2.45]	21.94 [11.75–33.46]	6.36 [4.15–9.46]	27.97 [18.69–39.14]	1.13 [0.75– 1.51]
Armenia	2.51 [2.09–2.98]	8.46 [7.06–10.11]	9.96 [8.06–11.85]	23.4 [18.96–27.89]	4.14 [3.48– 4.8]
Timor-Leste	0.15 [0.07–0.3]	4.62 [2.14–9.67]	0.57 [0.34–1.13]	6.39 [3.84–12.63]	1.09 [0.88– 1.29]
Qatar	0.4 [0.26–0.57]	30.1 [20.14–40.8]	3.19 [1.92–5.25]	25.32 [15.71–40.27]	–0.27 [–0.63 to 0.1]
Iraq	17.5 [10.7–24.8]	20.81 [12.74–29.41]	77.9 [50.27–111.45]	29.64 [19.4–42.24]	1.31 [1.12– 1.5]
Azerbaijan	3.19 [2.14–4.6]	5.5 [3.7–7.94]	8.4 [5.29–12.52]	7.12 [4.47–10.61]	1.49 [1.05– 1.93]
Iran (Islamic Republic of)	37.26 [24.28–50.35]	12.84 [8.5–17.52]	158.73 [101.03–188.59]	19.38 [12.38–23.03]	1.75 [1.6–1.89]
Jordan	3.96 [2.67–5.38]	26.87 [17.91–36.3]	19.41 [12.02–28.55]	23.48 [14.68–34.26]	–0.68 [–0.92 to –0.44]
Kuwait	2.3 [2.08–2.52]	34.74 [31.19–38.1]	6.8 [5.63–8.07]	19.4 [15.88–23.06]	–0.46 [–1.26 to 0.34]
Singapore	6.12 [5.68–6.56]	27.07 [25.13–29]	13.11 [11.84–14.44]	15.13 [13.62–16.67]	–1.74 [–1.86 to –1.62]
Kazakhstan	18.07 [14.09–21.82]	12.78 [10.01–15.38]	35.05 [29.28–41.36]	17.74 [14.84–20.9]	1 [0.75 –1.26]
Mongolia	0.6 [0.41–0.92]	4.97 [3.44–7.51]	2.39 [1.68–3.29]	8.4 [5.91–11.5]	2.01 [1.85–2.16]
United Arab Emirates	3.89 [2.08–7.23]	66.3 [37.04–119.89]	26.65 [18.14–38.64]	57.68 [41.04–77.14]	0.65 [0.26–1.05]

### 3.3 MM burden by age and sex

**FIGURE 3:**
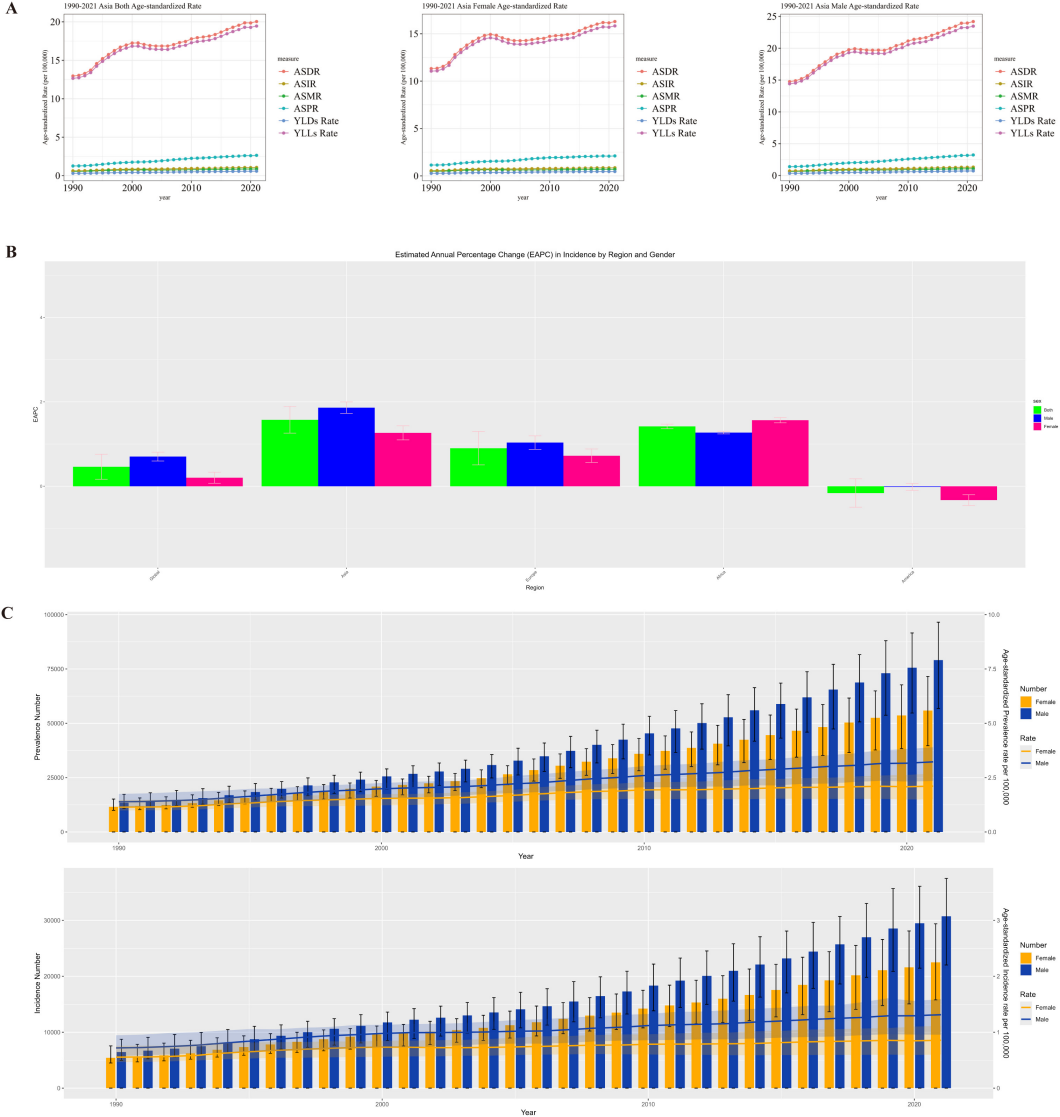
**(A)** Gender distribution of MM disease burden in Asia, **(B)** Asia EAPC in incidence by region and gender, **(C)** Asia distribution of the number of incidence and prevalence of MM patients in males and females.

From 1990 to 2021, Asia was the region with the most significant increase in the burden of MM incidence and the most prominent gender disparity, with a rapid rise in the incidence risk among men. In Africa and Europe, the incidence increased slowly in both genders, showing weak gender differences. In the Americas, the burden of MM incidence among women slightly decreased, and the overall growth stagnated, making it the only region in the world with a “negative growth in female incidence.” Driven by the high growth in Asia and the special situation in the Americas, the global trend showed a slow increase dominated by men. The patterns of gender-driven growth in the burden of MM incidence varied significantly across regions, as shown in Figures 3A,B.

The burden of MM in Asia (in terms of incidence and prevalence) continued to rise. Men had higher absolute case numbers and age-standardized rates than women, with a synchronized growth trend. This indicates that Asia needs to pay attention to the high burden of MM among men, while alerting to the trend of the overall disease burden worsening over time, and carry out targeted gender-stratified prevention, control, and disease surveillance, as shown in Figure 3C.

Asia needs to focus on the high burden of health-adjusted life years (HALY) loss caused by MM among men, while remaining vigilant against the cumulative increase in the overall disease burden over time. It is necessary to formulate stratified prevention and control strategies based on gender differences (e.g., strengthening early intervention for men and focusing on long-term disease management for women) to alleviate the continuous impact of MM on the healthy life span of the population.

In 2021, the epidemiological characteristics of MM in Asia exhibited a notable age-related trend. The prevalence rate increased with age, and both men and women reached the peak in the 70–74 age group, followed by a slight decrease in older age groups. This indicates that disease prevalence is concentrated in the elderly population, and as age increases further, the prevalence trend shows a certain decline.

**FIGURE 4:**
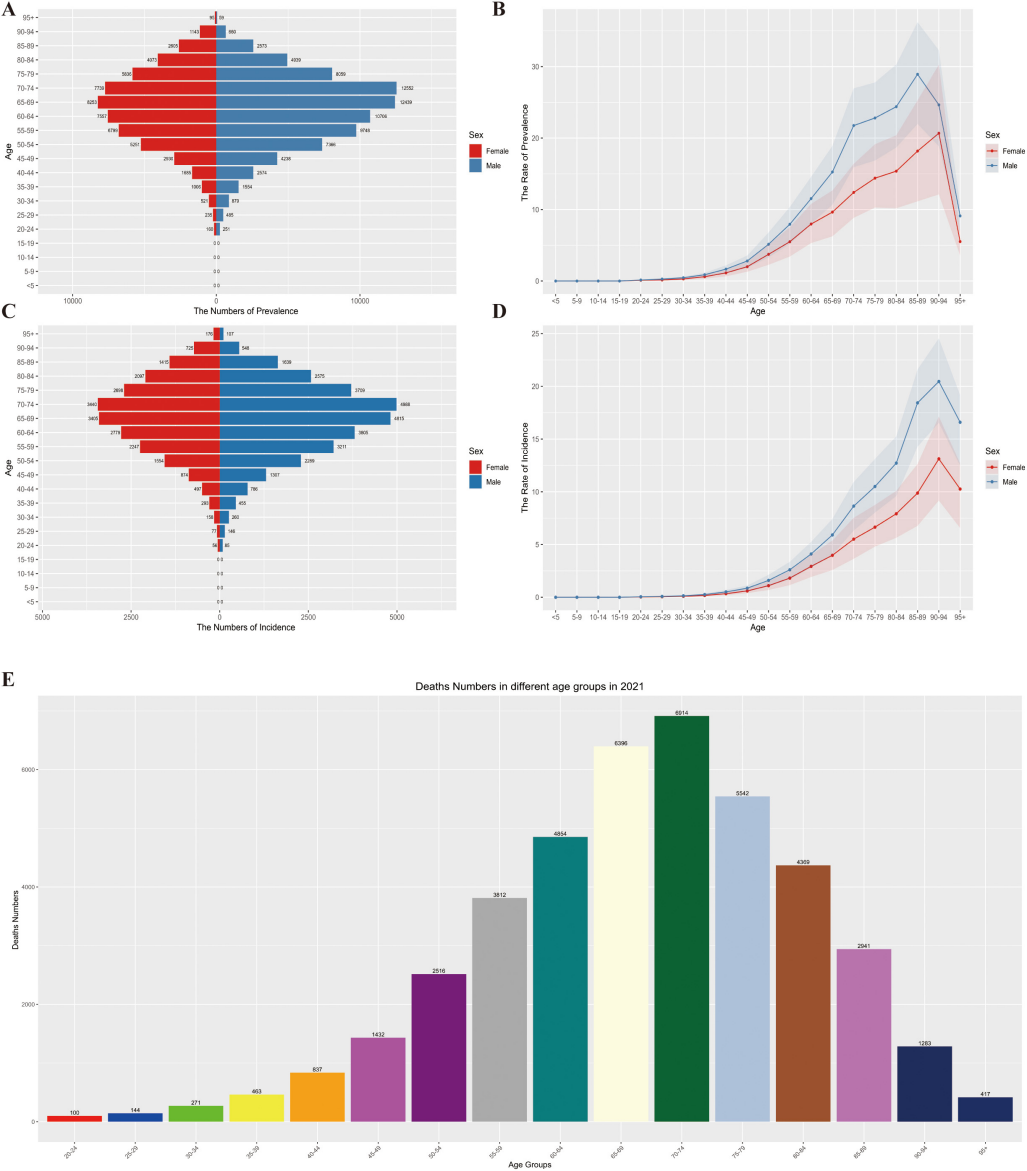
**(A)** Distribution of prevalence numbers by age group and gender, **(B)** trend of prevalence rate by age group and gender, **(C)** distribution of incidence numbers by age group and gender, **(D)** trend of incidence rate by age group and gender, **(E)** distribution of deaths by age groups in 2021.

The incidence rate gradually increased with age, following the same pattern in both genders, and also peaked in the 70–74 age group. This indicates that the older the age, the higher the risk of MM onset, and the elderly population is the “high-risk range” for MM incidence, as shown in Figures 4A–D.

The number of deaths gradually increased with age, with consistent trends in both men and women, and remained at a high level in the elderly stage (e.g., the 70–74 age group and older age groups). This demonstrates that MM has a continuous and significant impact on the death of elderly patients, the older the age, the heavier the burden of death, as shown in Figure 4E.

Overall, the prevalence, incidence, and mortality of MM in Asia are all closely related to age. The elderly population (especially those aged 70–74 and above) is the core group bearing the disease burden. The impact of MM accumulates with age, and its threat to the health of elderly patients is persistent and prominent.

### 3.4 Correlation between HDI and EAPC

**FIGURE 5:**
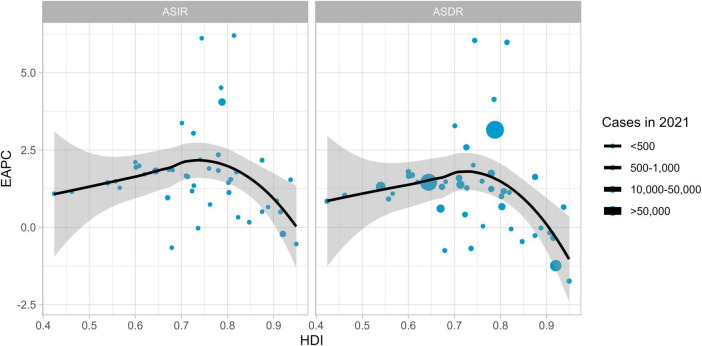
The evolution between HDI and EAPC in 2021.

In 2021, the trends of ASIR and ASDR of MM showed corresponding changes with the national Human Development Index (HDI) (ASIR correlation = –0.08, ASDR correlation = –0.20); (see Figure 5). As the HDI increased, the EAPC initially showed an upward trend, reached a peak when the HDI reached a certain level, and then the EAPC began to decline. The shaded areas represent confidence intervals. Larger data points (corresponding to higher case numbers) are scattered across different HDI and EAPC ranges, indicating that, in terms of ASIR and ASDR, the association between HDI and EAPC follows a similar pattern, while the number of cases adds another layer of variation to this relationship.

### 3.5 Frontier analysis based on age-standardized DALYs

**FIGURE 6:**
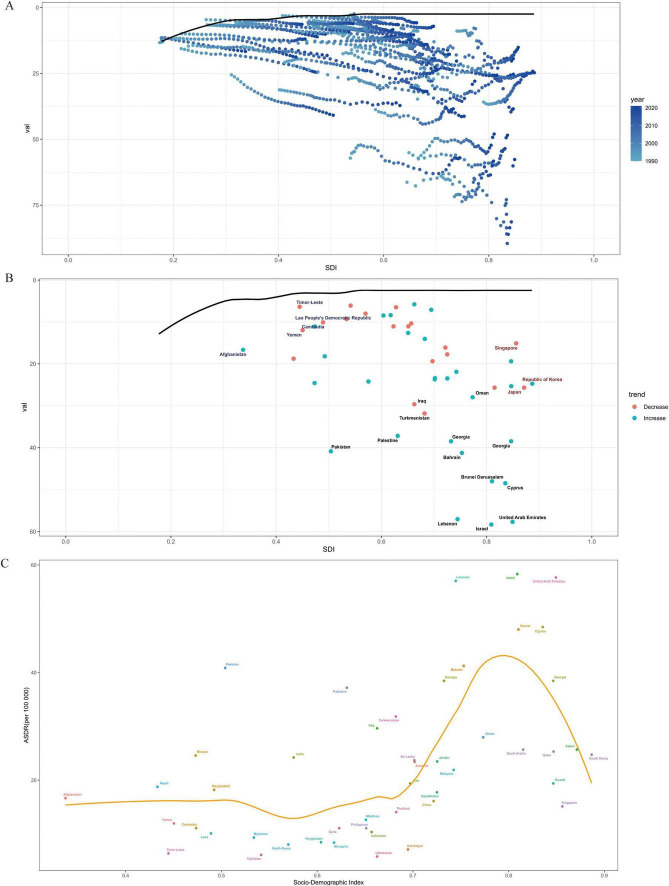
Frontier analysis of ASDR for MM 2021. **(A)** Distribution relationship between SDI and a certain indicator in different years, **(B)** distribution of SDI and a certain indicator with trends in different countries, **(C)** relationship between SDI index and ASDR.

To explore the association between disease burden (measured by DALYs) and regional development status (SDI) across 48 Asian countries, a frontier analysis was conducted using longitudinal data from 1990 to 2021 (as shown in Figure 6). The constructed frontier boundary can identify the minimum age-standardized DALY rate achievable at a specific SDI level. The analysis revealed an inverted U-shaped relationship between the disease burden efficiency gap and development status: countries with high SDI values had the smallest efficiency gaps, while those with medium SDI values exhibited the most significant gaps.

Among the 48 Asian countries, Grenada, New Zealand, Uruguay, and Saint Lucia are among those with great potential to narrow the gap. Notably, outstanding performers are not limited to developed countries. Within Asia, countries with low SDI ( < 0.2), such as some war-torn and impoverished nations, showed small actual disease burden gaps. In contrast, certain Asian countries with high SDI ( > 0.7), including Japan and South Korea, did not perform as expected in terms of SDI, leaving room for improvement in disease burden efficiency. This suggests that the relationship between development level and disease burden management efficiency is not strictly linear, and targeted optimization of medical resources and prevention/control strategies is necessary to narrow the disease burden gap between countries at different development stages.

### 3.6 Disease burden projections for MM

By 2050, the burden of MM in Asia (measured by incidence and DALYs) will continue its growing trend. The prevalence and incidence rates for both men and women are projected to first increase and then stabilize. Whether considering the ASIR or the ASDR, the trends are consistent between genders. This suggests that the overall pressure of MM prevention and control in Asia will persist over time, and the disease’s impact on population health will become long-term.

**FIGURE 7:**
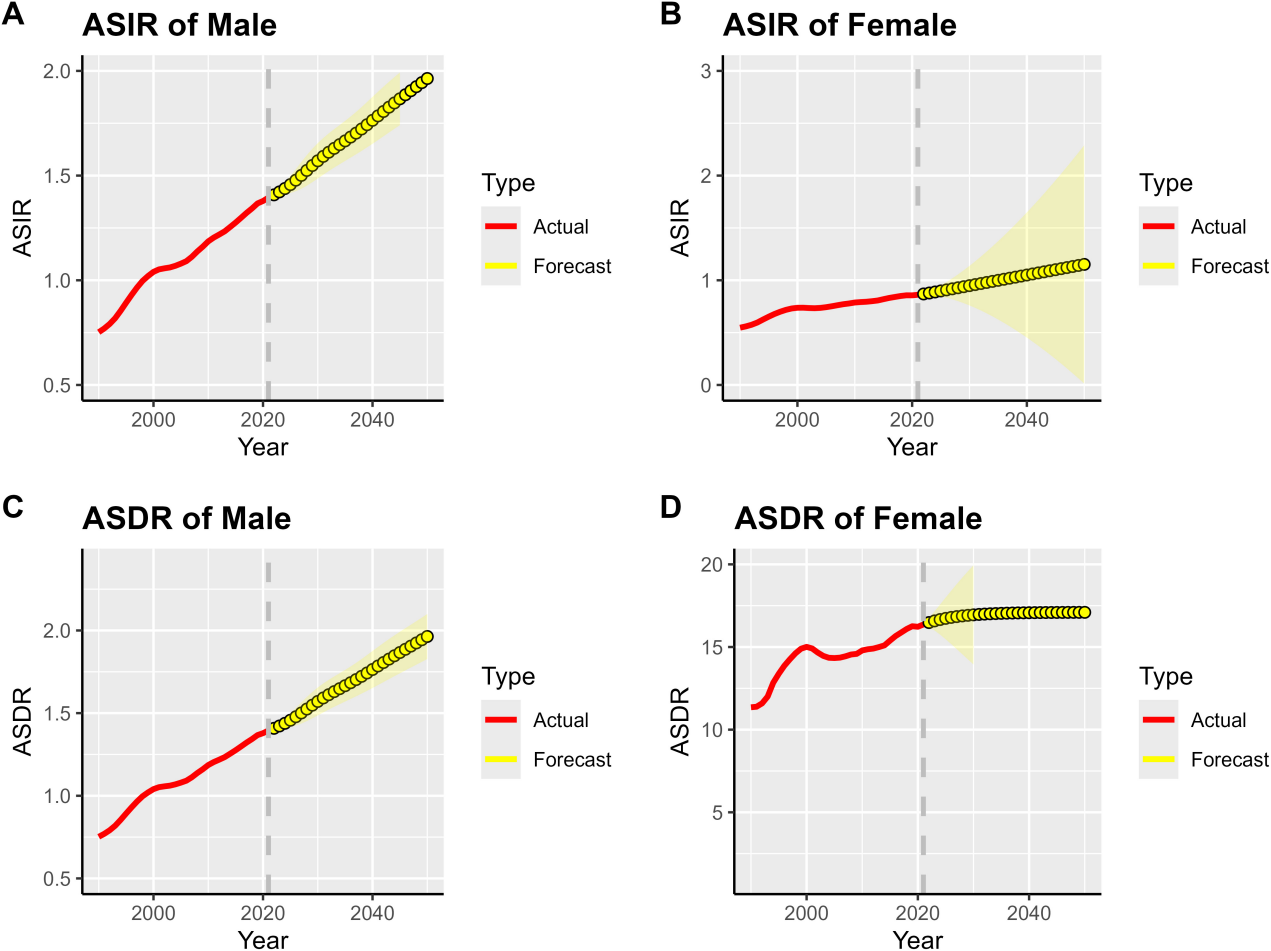
Projected global MM burden, 1990–2050. **(A)** ASIR of Male, **(B)** ASIR of Female, **(C)** ASDR of Male, **(D)** ASDR of Female.

The ASIR and ASDR in men are significantly higher than those in women. As shown in Figure 7, the growth slopes of ASIR and ASDR in men are steeper, and both the projected peak values and stable values are higher than those in women. This suggests that men in Asia are more severely affected by MM and should be the key population for disease prevention and control. Targeted efforts are needed to strengthen screening, diagnosis, treatment, and health management for men.

Over time, the prediction interval (yellow shaded area) gradually widens, reflecting inherent uncertainties in the long-term projection of MM disease burden in Asia. These uncertainties stem from multiple factors, including changes in population structure, advancements in medical technology, and adjustments to prevention and control strategies. Therefore, it is necessary to continuously conduct disease surveillance, deepen research, and dynamically collect data to optimize existing prediction models. This will provide more reliable evidence for future medical resource planning in Asia (e.g., male-specific screening programs, preferential allocation of diagnosis and treatment resources) and enable precise responses to the challenges posed by the MM disease burden.

## 4 Discussion

This study, based on the GBD 2021 database and supplemented with longitudinal data from 1990 to 2021, conducted a systematic analysis of the disease burden of MM across 48 Asian countries, examining four core dimensions: incidence, prevalence, mortality, and DALYs. Meanwhile, it integrated methods such as trend analysis, gender stratification, regional comparison, and ARIMA model prediction to comprehensively reveal the spatiotemporal evolution patterns, driving factors, and future challenges of MM burden in Asia, thereby providing a scientific basis for formulating precise prevention and control strategies within the region.

By 2021, the burden of MM in Asia had reached a substantial scale. There were 53,218 new cases (35.8% of the global total), representing an increase of more than 348% from 1990 and 3.3 times the global average growth rate; 134,965 prevalent cases (34.2% of the global total), a rise of 439.2% and making Asia the only region among the four continents with a prevalence growth rate of over 400%; 42,292 deaths (36.3% of the global total), which surpassed Europe for the first time to make it the continent with the heaviest MM mortality burden worldwide; and 1,030,207 DALYs (39.7% of the global total), an increase of 280.8% that is far higher than that of the Americas (91.2%) and Europe (62.8%).

From 1990 to 2021, Asia’s ASIR of MM rose from 0.63 to 1.07 per 100,000 (EAPC = 1.59), ASPR from 1.26 to 2.64 per 100,000 (EAPC = 2.49), and ASMR from 0.56 to 0.86 per 100,000 (EAPC = 1.18). All standardized indicators grew faster than the global average, making Asia the core driver of the global increase in MM burden.

Based on the ARIMA model prediction, the burden of multiple myeloma (MM) in Asia is expected to follow a “first rise and then stabilize” trajectory by 2050. The prevalence and incidence rates for both men and women are projected to show an upward trend, and then gradually stabilize after 2040. However, the peak values and stable-phase levels of all indicators for men will be significantly higher than those for women. Meanwhile, as the prediction period extends, the width of the prediction interval for men’s indicators is significantly larger than that for women, reflecting higher uncertainty in the long-term evolution of MM burden among men. Targeted efforts are therefore needed to strengthen dynamic monitoring for this group.

The MM mortality peaks for both men and women in Asia are concentrated in the 70–74 age group: Deaths in this age group account for 22.3% of the total deaths among men and 19.8% among women. Notably, this mortality peak occurs 5 years earlier than the incidence peak (75–79 age group), suggesting that elderly patients face a higher risk of death during the disease progression stage. It is worth noting that a reversal of gender disparity emerges in the elderly group aged 90 and above: The proportion of MM cases (including incidence, prevalence, and mortality) among women reaches 11.2%, which is significantly higher than that among men (6.8%). This phenomenon is closely related to the overall shorter survival period of male patients (with an average survival period 2.3 years shorter than that of women) and also reflects that elderly women need to cope with a more prolonged disease burden in the long-term management of MM.

The core driving factors behind the gender disparity include three aspects. First, the smoking and alcohol consumption rates among men in Asia are higher than those among women, and both smoking and alcohol consumption have been confirmed to be positively correlated with the risk of MM onset (32). Second, the prevalence of obesity in men is higher than that in women, and the chronic inflammatory state caused by high BMI can accelerate the abnormal proliferation of plasma cells, thereby increasing the risk of MM (33). Third, genetic and physiological differences. Men have lower expression of X chromosome-related immune regulatory genes and weaker immune surveillance, making multiple myeloma (MM) cells more likely to evade immune attacks. This also explains why MM in men is mostly diagnosed at the intermediate or advanced stage (34).

Within Asia, the MM burden presents a pattern characterized by “dominance in East and South Asia, and high growth rate in Central Asia.”

East Asia (China, Japan, South Korea, etc.): In 2021, the number of new MM cases accounted for 38.2% of Asia’s total. China, Japan, and South Korea together contributed 50.5% of all MM cases in Asia. Additionally, the EAPC of ASIR (2.13%) in this region was significantly higher than the regional average, making East Asia the “core bearing area” of Asia’s MM burden. South Asia (India, Pakistan, Bangladesh, etc.): New cases in this region made up 32.7% of Asia’s total. India ranked second in Asia in terms of MM incidence, mortality, and DALYs. Notably, the misdiagnosis rate of MM in this region is extremely high, meaning the actual disease burden may be underestimated. Central Asia (Kazakhstan, Uzbekistan, etc.): Although the absolute value of the MM burden is relatively low, the growth rate is rapid, with an ASIR EAPC of 1.92%. Among them, Kyrgyzstan (3.37%) and Turkmenistan (6.12%) have the two highest growth rates in Asia. This phenomenon is primarily linked to the significant advancements in medical diagnostic capabilities in this region over the past few years. West Asia (Turkey, Iran, etc.): A divergence has emerged, with “stable to declining trends in high-income countries and rapid increases in low- and middle-income countries.” For instance, the ASIR EAPC of Israel (0.50%) is significantly lower than that of Georgia (6.20%). Southeast Asia (Indonesia, Thailand, etc.): The MM burden shows a steady upward trend, with an ASIR EAPC of 1.64%. Indonesia has the highest MM burden in this region.

The level of socioeconomic development (measured by SDI and HDI) also exerts an impact on the MM burden in Asia. High-income countries (e.g., Japan, South Korea, and Singapore) have well-established healthcare systems, with an MM diagnosis rate of over 85%, so their ASIR data are closer to the actual incidence level. In contrast, low-income countries (e.g., Afghanistan, Yemen) have a diagnosis rate of < 30%, with a large number of “hidden cases” (undiagnosed cases). This leads to a significantly lower ASIR (0.4–0.6 per 100,000) than the regional average (1.07 per 100,000), resulting in a severe underestimation of the actual disease burden.

As socioeconomic status decreases, the average age at death of MM patients decreases significantly: the average age at death is 72.5 years in high-income countries, 68.3 years in middle-income countries, and only 63.1 years in low-income countries. This phenomenon is mainly related to the accessibility of targeted drugs (35).

Asia is the region with the largest population and the fastest aging rate globally. In 2021, the proportion of the population aged 65 and above reached 9.6%, representing a 5.2 percentage point increase from 1990. As an “elderly disease,” the incidence risk of MM in people aged over 65 is 8.3 times that of people under 65. Meanwhile, the population size effect of China (1.41 billion) and India (1.39 billion) further amplifies the burden: in 2021, the two countries together accounted for 60.2% of new MM cases, 62.3% of deaths, and 65.4% of DALYs in Asia, making them the “core drivers” of Asia’s MM burden. In addition, the extension of life expectancy in Asia (rising from 61.5 years in 1990 to 73.2 years in 2021) has also led to a longer “disease survival period” for MM, directly increasing the number of prevalent cases and the DALY burden (36).

Obesity and related metabolic diseases have become important risk factors for MM incidence in Asia. In 2021, the prevalence of adult obesity in Asia reached 16.4%, a 2.3-fold increase compared with 1990, and high BMI further induces the occurrence of MM (37). Moreover, in certain parts of Asia (e.g., northern China, northwestern India), high-sodium and high-fat diets, as well as sedentary lifestyles, are prevalent, resulting in a significantly higher prevalence of metabolic syndrome compared to the global average. Metabolic syndrome can accelerate the malignant transformation of plasma cells by inducing chronic inflammation, insulin resistance, and other mechanisms, thereby further increasing the risk of MM (38).

The improvement in MM diagnostic capabilities in Asia (e.g., increased popularity of serum protein electrophoresis and bone marrow biopsy) has led to the continuous detection of “hidden cases,” driving up the ASIR and ASPR. On the other hand, advances in treatment technologies (e.g., the application of targeted drugs and immunotherapy) have slowed the growth rate of mortality. This has resulted in a lower EAPC of MM ASMR (1.18%) in Asia compared to that of ASIR (1.59%) and ASPR (2.49%), forming a pattern of “rapid increase in incidence but slow increase in mortality” and also leading to the continuous accumulation of prevalent cases.

Although predictions indicate that the growth rate of Asia’s MM burden will stabilize after 2040, the ASIR for both men and women is expected to continue an upward trend by 2050. Therefore, it is necessary to establish a three-level “national-regional-global” MM surveillance network, with a focus on covering regions with rapid growth and insufficient data (such as Central Asia and Southeast Asia). This network should dynamically supplement case report data to reduce prediction uncertainty. Meanwhile, it is essential to optimize the ARIMA model by incorporating variables such as the rate of population aging, trends in obesity prevalence, and investment in medical resources. This optimization will improve the accuracy of long-term predictions and provide more reliable evidence for medical resource planning (e.g., bed quantity and drug reserves) (39).

In response to the characteristic that the disease burden is heavier among men, it is necessary to launch MM screening pilots targeting high-risk groups (e.g., men over 50 years old, men who smoke or are obese) and promote a combined screening protocol of “serum protein electrophoresis and complete blood count.” Meanwhile, it is essential to strengthen health education for men to reduce their exposure to risk factors such as smoking and harmful alcohol consumption.

Regarding the rising proportion of elderly female MM cases, strategies for managing MM in elderly women need to be optimized, for instance, adjusting the dosage of targeted drugs, enhancing nutritional support, and preventing complications to extend the survival period of elderly patients.

To tackle the imbalance in the allocation of MM diagnosis and treatment resources across Asia, it is recommended to establish a three-level collaborative mechanism involving countries with different socioeconomic development levels. High-income countries should support low-income countries through the dual pathways of “technology transfer and drug donation,” by establishing equipment training systems and closed-loop drug distribution networks, thereby achieving a shift from pure resource infusion to sustainable capacity building. Middle-income countries such as India and Indonesia ought to develop regional MM diagnosis and treatment hubs, which can radiate to neighboring areas through the hierarchical management framework of “primary-level screening and centralized diagnosis and treatment.” This framework is expected to significantly reduce the misdiagnosis rate of MM, thereby minimizing the incremental disease burden caused by delayed treatment and ultimately forming a comprehensive MM diagnosis and treatment network covering the entire Asian region.

Asian countries need to construct full-chain prevention and control system for MM. Specifically, screening for Monoclonal Gammopathy of Undetermined Significance (MGUS) should be integrated into the routine physical examination of individuals aged 45 years and above, with enhanced screening frequency for high-risk groups. A regional MM case information sharing database should be established to enable dynamic follow-up and risk early warning, which will substantially improve screening coverage and continuously increase the early diagnosis rate of MM. Meanwhile, an Asian MM new drug access collaboration mechanism should be established to introduce novel therapeutic agents, including proteasome inhibitors, immunomodulatory drugs, and monoclonal antibodies (40). This initiative aims to enhance the standard of MM diagnosis and treatment, and narrow the gap in therapeutic outcomes between Asian countries and developed nations.

The implementation of “co-prevention of multiple diseases” based on metabolic disease management systems represents a crucial public health strategy for reducing MM incidence risk. Primary healthcare institutions should conduct simultaneous MM risk assessments for patients with obesity or diabetes, incorporate metabolic indicators into the high-risk scoring system, and provide integrated interventions combining personalized weight management (encompassing dietary guidance tailored to metabolic characteristics and exercise prescriptions adapted to physical fitness levels) and targeted MM prevention strategies (including regular MGUS screening and health education on MM-related risk factors) for high-risk populations. Governments should introduce policies to restrict the promotion of high-sugar and high-fat foods, and expand the construction of public fitness facilities. These measures will reduce the prevalence of obesity at the source, thereby decreasing the incidence risk of MM.

Overall, through the formulation of public health policies and the allocation of resource frameworks including hierarchical resource allocation, full-chain prevention and control, and integrated management of comorbidities, the equity in MM diagnosis and treatment can be further enhanced. In the long run, such a policy framework is expected to reduce the incidence growth rate of new MM cases in Asia, serving as a reference for the regional prevention and control of MM worldwide.

Like other GBD studies, this research also has certain limitations. First, the GBD model relies on homogenizing assumptions such as “regional extrapolation” and “trend fitting.” It often estimates the burden with reference to regional average characteristics or disease patterns in high-income regions, which may lead to deviations in the burden estimation of low-income regions and fail to reflect the actual situation. Second, MM has a different molecular subtype, which show significant differences in clinical progression and prognosis, but the GBD does not provide subtype-specific disease burden analysis. During the COVID-19 pandemic, delays occurred in MM screening, diagnosis, and treatment, which may affect the accuracy of data from 2020 to 2021 and the judgment of long-term mortality trends. Both the clinical heterogeneity of MM and the problem of underdiagnosis and misdiagnosis in regions with low SDI may underestimate the actual disease burden.

Despite these limitations, the present study offers valuable insights into understanding the prevalence trends of MM and future changes on a global scale. The results emphasize the importance of preventive strategies, such as promoting healthy weight management and lifestyle interventions to reduce disease risk, and suggest more targeted screening and management measures for at-risk populations. These findings may provide a scientific basis for developing more effective MM prevention and management strategies, especially in areas with a high disease burden and relatively limited healthcare resources.

## 5 Conclusion

Based on the GBD 2021 database, this study systematically analyzed the spatiotemporal evolution and driving factors of the MM disease burden in Asia from 1990 to 2021, and clearly identified its core characteristics of “continuous expansion in scale, significant gender disparity, and prominent regional differences.” As the core driver of the global growth in MM burden, Asia bears greater pressure on men, the elderly population, and regions in East/South Asia. Population aging, lifestyle-related risks, and uneven distribution of medical resources have collectively shaped the pattern of the MM disease burden.

In the future, the prevention and control of MM in Asia will need to confront the challenges of long-term growth. It is necessary to consolidate the data foundation through precise monitoring, address the issues of gender and regional disparities with targeted strategies, compensate for the shortage of medical resources through international collaboration, and integrate metabolic management to advance the prevention and control front. Only through multi-stakeholder collaboration and targeted measures can we promote the transformation of the prevention and control model from “passively responding to the disease burden” to “proactively shaping a healthy future,” gradually reduce the impact of MM on the health of Asian populations, provide Asian experiences and solutions for global disease burden governance, and contribute to the achievement of health equity and sustainable development goals.

## Data Availability

The original contributions presented in the study are included in the article/Supplementary material, further inquiries can be directed to the corresponding authors.

## Footnotes

1
http://ghdx.healthdata.org/gbd-results-tool

2
http://hdr.undp.org/en/data
